# Exploring the Relationship between Health Literacy and Depression among Individuals with Chronic Conditions: Insights from the KCHS

**DOI:** 10.3390/healthcare12191927

**Published:** 2024-09-26

**Authors:** Eungyeong Kim

**Affiliations:** Department of Nursing, Kunsan National University, Gunsan 54150, Republic of Korea; egkim@kunsan.ac.kr; Tel.: +82-63-469-1994

**Keywords:** health literacy, depression, chronic disease

## Abstract

**Objectives:** This study, utilizing data from the 2021 Korean Community Health Survey (KCHS), aimed to investigate the role of health literacy in mitigating depression among individuals with chronic diseases, particularly focusing on how sociodemographic and subjective factors influence this relationship. **Methods:** With a sample of 77,133 subjects primarily dealing with hypertension and diabetes, the study employed various analytical tools to identify factors associated with an increased risk of depression. **Results:** These factors included marital status (OR 1.47, 95% CI 1.40–1.54), residential district (OR 1.43, 95% CI 1.39–1.50), unemployment (OR 1.47, 95% CI 1.41–1.54), enrollment in the national basic living security program (OR 1.50, 95% CI 1.40–1.62), unmet healthcare needs (OR 2.32, 95% CI 2.14–2.51), fair subjective health status (OR 1.74, 95% CI 1.63–1.90), poor subjective health status (OR 4.33, 95% CI 4.05–4.63), and stress (OR 4.56, 95% CI 4.36–4.77). Notably, individuals with higher health literacy showed a significantly lower susceptibility to depression (OR 0.74, 95% CI 0.68–0.75), underscoring the importance of health literacy as a critical factor in mental health outcomes. **Conclusions:** Such initiatives could serve as effective preventive measures against depression in this vulnerable population.

## 1. Introduction

Chronic diseases contribute significantly to the annual death toll in South Korea, accounting for 80% of all deaths [1]. These diseases have been recognized as a major concern by the Organisation for Economic Cooperation and Development (OECD) due to the high associated death rates [1]. Managing chronic diseases requires a focus on symptom relief and complication prevention, as complete cures may not always be achievable. Proper lifestyle choices, adherence to medication regimens, and regular medical checkups are crucial for disease monitoring and management [2].

In the context of chronic disease management, health literacy has been recognized as an essential intervention [3]. Health literacy encompasses an individual’s capacity to acquire, process, comprehend, evaluate, and utilize health information and services essential for making informed decisions regarding health [4]. Health literacy empowers individuals to manage their symptoms and complications independently, while also contributing to overall health improvement and addressing social and environmental health issues [5]. Particularly during the COVID-19 pandemic, the ability to understand and apply health information has become even more critical for enhancing individual health outcomes [3].

Various studies have shown the impact of health literacy on different aspects of chronic disease management. For instance, research on stroke patients demonstrated that higher health literacy levels correlated with more effective implementation of re-habilitation [6]. Additionally, health literacy has been identified as a significant factor influencing health behaviors among stroke patients [7]. Similar findings were observed in a study involving hemodialysis patients, where higher health literacy levels were associated with more proactive patient role behavior [8]. Furthermore, among vulnerable populations with chronic diseases, higher health literacy was linked to improved self-nursing competency [9].

According to previous studies, approximately 12% of South Korean adults report experiencing both depression and chronic diseases [10]. Patients with chronic disease face an elevated risk of depression due to various factors such as the burden of their condition, challenges in treatment adherence, fear of relapse, and the presence of persistent symptoms [10]. In South Korea, these challenges are exacerbated by persistently high rates of suicide and depression, coupled with a widespread reluctance among the population to seek mental health services [11]. In response to these pressing realities, there has been a growing emphasis on preventive approaches to mental well-being. Recent research in the social sciences has increasingly focused on health literacy and its impact on mental health. Health literacy refers to the ability to acquire, comprehend, and assess medical information to make informed decisions related to healthcare [12]. Previous studies have highlighted the negative correlation between mental health and lower health literacy levels [12]. Several studies have also indicated a positive effect of health literacy on preventing and treating depression [13]. Individuals with higher health literacy tend to experience lower levels of depression, and some studies have even identified depression as a significant variable influencing health literacy ability [12,13,14].

Despite recognizing the critical relationship between health literacy and mental health, there remains a notable scarcity of domestic studies investigating this connection in South Korea. Given the significant relationship between health literacy and mental health, this study aims to contribute to the understanding of depression prevention strategies by examining the influence of sociodemographic and subjective factors on health literacy among chronic patients in South Korea. Previous studies on health literacy comprehension have faced challenges due to data collection through direct surveys, leading to limited geographical coverage and constrained generalizability to the entire population nationwide [13,14,15]. To bridge these gaps, this research seeks to illuminate depression prevention strategies by investigating the factors influencing the health literacy of chronic patients. Leveraging large-scale, nationwide data obtained from the Korean Community Health Survey (KCHS), this analysis aims to provide foundational insights into the importance of health literacy in managing depression among this population. While the study delves into the intricate relationship between mental health and health literacy, the nuanced interplay within the challenging contextual realities individuals encounter is acknowledged. Cultural beliefs, socioeconomic status, and access to resources undoubtedly shape the effectiveness of health literacy interventions in promoting mental well-being [11]. Therefore, this study endeavors to consider these complexities and aims to offer insights that can inform tailored strategies for addressing mental health challenges across diverse populations. In this study, I aim to contribute to creating conditions in which individuals can recognize and act upon their innate agency. By investigating the relationship between health literacy and depression among chronic patients, we seek to shed light on factors that may facilitate or hinder individuals’ ability to make informed decisions about their mental health. Through this research, I hope to identify strategies and interventions that can support individuals in recognizing and utilizing their agency to manage their mental well-being effectively.

## 2. Materials and Methods

### 2.1. Data and Sample

This study utilized secondary data from the 2021 Korea Community Health Survey (KCHS), conducted nationwide in South Korea. The KCHS is a comprehensive, standardized survey conducted annually by public health centers since 2008. Its primary aim is to generate regional health statistics essential for local health care planning and to produce indicators that support evidence-based health projects.

#### 2.1.1. Sample Extraction

The sampling frame was developed by integrating the resident population data from the Ministry of the Interior and Safety with housing data from the Ministry of Land, Infrastructure, and Transport. This integration provided a comprehensive list of the entire population. Sampling points were determined based on housing types within administrative units (e.g., neighborhoods, villages). The probability of selection for each point was proportional to the number of households, ensuring that larger areas with more households had a higher probability of being selected. Within the selected sampling points, households were identified and chosen using systematic sampling methods. This approach involved selecting households in a systematic manner to ensure that the sample was representative of the population within each sampling point.

#### 2.1.2. Survey Targets and Timing

Survey Population: Adults aged 19 and older residing in the sampled households at the time of the survey were included in the study. Data collection occurred from 16 August 2021 to 31 October 2021.

#### 2.1.3. Survey Methodology

Trained interviewers conducted face-to-face interviews with selected households using the computer-assisted personal interviewing (CAPI) system. This method allows for real-time data entry and reduces the risk of data entry errors. The survey was administered through direct, in-person interviews to ensure accurate data collection and to address any questions or clarifications needed by respondents. While specific response rates are detailed in the official survey documentation, the KCHS typically achieves high response rates due to its structured methodology and comprehensive coverage. For further details on the sampling methodology and survey procedures, please refer to the official KCHS documentation available on the KCHS website [16].

### 2.2. Measurement

#### 2.2.1. Chronic Disease

The study specifically focused on patients diagnosed with diabetes mellitus and hypertension, as these are the chronic conditions included in the 2021 Korea Community Health Survey (KCHS). I chose to include only those with these diseases to align with the data provided by the KCHS and to ensure a consistent and relevant sample for the analysis. Regarding the inclusion criteria of patients on medication, this decision was made to target individuals with confirmed diagnoses and active management of their conditions. Medication usage serves as an indicator of ongoing medical supervision and treatment, which is essential for assessing the relationship between health literacy and depression. By including only those on medication, I aimed to ensure that the participants had a recognized and managed chronic condition, thereby enhancing the accuracy and relevance of the findings. This approach helps in focusing on individuals who are actively dealing with their chronic diseases and might be more representative of the broader population facing similar health challenges.

#### 2.2.2. Health Literacy (HL)

The KCHS measures health literacy (HL) based on the Behavioral risk factor surveillance system (BRFSS) framework [16]. In this study, HL was assessed using two questions from the 2021 KCHS: “How difficult is it for you to understand information that doctors, nurses, and other health professionals tell you?” (to assess understanding of oral information) and “You can find written information about health on the internet, in newspapers and magazines, and in brochures in the doctor’s office and clinic. In general, how difficult is it for you to understand written health information?” (to assess understanding of written information). Responses were rated on a 4-point Likert scale: very difficult (score = 1), somewhat difficult (score = 2), somewhat easy (score = 3), and very easy (score = 4). Higher scores indicate a higher level of health literacy. The HL score was calculated by averaging the scores from the two questions, and participants were categorized into two groups based on a cutoff point: <3 (low health literacy) and ≥3 (high health literacy).

While numerous studies have explored the impact of “mental health literacy”, a concept specific to understanding mental health information and managing mental health conditions, this study addresses a broader and distinct aspect of health literacy. Unlike mental health literacy, which focuses exclusively on knowledge and understanding related to mental health, this research investigates general health literacy and its relationship with depression in individuals with chronic conditions. The originality of this investigation lies in its examination of health literacy as a general construct—encompassing both oral and written health information understanding—and its impact on depression among chronic disease patients, particularly within the context of South Korea. By utilizing a comprehensive approach to health literacy assessment and focusing on its general effects rather than specific mental health literacy, this study provides new insights into how overall health literacy influences mental health outcomes. This broader perspective is significant, as it highlights the potential for improving general health literacy to enhance mental health management and outcomes among individuals with chronic conditions, thus offering a novel contribution to the field.

##### Reliability and Validity

Given that the health literacy measurement used in this study differs from more commonly used international scales, it is crucial to address its reliability and validity. While the specific reliability and validity of these two questions have not been extensively validated in the literature, they were selected based on their alignment with the BRFSS framework [16], which has been employed in various health literacy assessments. The use of a simple two-item measure is supported by prior research indicating that brief assessments can provide useful insights into general health literacy levels [17].

##### Basis for the Cutoff Value

The choice of a cutoff value of 3 was informed by practical considerations rather than extensive empirical validation. In this study, a score of <3 was designated as indicative of low health literacy, while ≥3 was considered high health literacy. This dichotomization aims to identify individuals who may have more pronounced difficulties with health information, thereby facilitating targeted interventions. While this cutoff is not derived from a specific empirical study, it is intended to serve as a practical screening tool to distinguish between varying levels of health literacy [18].

##### Purpose of Screening

The term “cutoff” in this context is used to establish a screening threshold for categorizing participants into low- or high-health-literacy groups. The purpose of this screening is to identify individuals who may benefit from additional support or educational resources related to health information. By applying this dichotomous classification, the study aims to highlight differences in health outcomes, such as depression, based on levels of health literacy.

#### 2.2.3. Depression

Depression was measured using the diagnostic score of the Patient Health Questionnaire-9 (PHQ-9) [18]. The questionnaire consists of nine items, each rated from 0 to 3. Thus, the total score can range from 0 to 27 (0 represents the lowest level of depression, and 27 represents the highest level of depression). In this study, the classification was based on a previous study that defined depression as “yes” for PHQ-9 scores greater than 5 and “no” for scores less than or equal to 5 [19]. Cronbach’s coefficient α of development was 0.84 [18], and this time was 0.80.

#### 2.2.4. Control Factors

Control factors included age, sex, marital status, residential district, education level, job, recipients of the national basic living security program, subjective health, and stress. Age was classified into two categories (<65/≥65). Marital status was having a spouse (yes/no). Residential districts were classified into urban and rural. The classification into urban or rural areas was based on the subjects’ residential addresses and the corresponding administrative districts. The classification of education levels was modified based on South Korea’s educational structure, which includes elementary school, middle school, high school, and university or higher education (>12 years). Therefore, in this study, the education level was divided into two categories, “≤middle school” and “>middle school”, and job was classified into two categories, “unemployed/employed”. The National Basic Livelihood Security Program is a social welfare initiative in the Republic of Korea that provides financial assistance to individuals whose recognized amounts of income fall below the minimum cost of living threshold. The recipients of the national basic livelihood security program were classified as “yes/no”. Unmet healthcare need is defined as a situation where an individual answers yes to the question, “Have you been unable to go to a hospital in the last year when you wanted to?” The questions related to subjective health and stress were original questions included in the KCHS survey. Subjective health was measured using the question “When you think about yourself, how do you feel about your health?” Responses ranged along a 5-point scale from “very good” to “very bad”. They were reclassified into “good/fair/bad”. Stress was measured using the question “How much stress do you feel in your everyday life?” Responses ranged along a 4-point scale from “feel a lot” to “hardly feel it”. They were reclassified into two categories, “yes/no”.

### 2.3. Statistical Analysis

Data analysis was performed using IBM SPSS Statistics version 21.0 for Windows (IBM Corporation, Armonk, NY, USA). Descriptive statistics were used to summarize smartphone overuse and subject characteristics, presented as frequencies and percentages. Chi-squared statistics were used in bivariate analyses to examine associations between smartphone overuse and subject characteristics. For multivariate analysis, logistic regression was conducted to identify factors affecting smartphone overuse. Results are presented as odds ratios (ORs) with 95% confidence intervals (CIs), and a *p*-value of <0.05 was considered statistically significant.

Given the large and robust nature of the dataset, missing data for each variable were estimated using the multiple imputation method to avoid bias and loss of statistical power. Multiple imputation was performed to account for the different patterns and amounts of missing data across variables, ensuring the validity of the logistic regression results. Five imputed datasets were generated and pooled for final estimates, in line with best practices for handling missing data in large datasets.

## 3. Results

Among the study subjects, 63.1% had hypertension, 22.8% had both hypertension and diabetes, and 14.1% had diabetes only. In terms of general characteristics, 59.9% of participants were aged 65 or older. Gender distribution was fairly balanced, with 53.5% women and 46.5% men. Most participants (68.8%) were married, and about half (53.4%) resided in rural areas. Regarding education, 55.2% had education below middle school level, and 53.1% were employed. Additionally, 6.5% were recipients of basic living support and 4.7% had unmet healthcare needs. Subjective health was mostly reported as “fair” (44.6%), with 19.6% experiencing stress. Health literacy scores ≥3 were found in 60.6% of the subjects, and 18.0% exhibited symptoms of depression (Table 1).

Differences in health literacy and depression according to chronic diseases are summarized in Table 2. A higher prevalence of health literacy scores < 3 was observed among those with both hypertension and diabetes (41.0%) compared to other groups (χ^2^ = 98.00, *p* < 0.001). Similarly, the prevalence of depression was significantly higher in this group (χ^2^ = 169.86, *p* < 0.001).

Table 3 details the differences in depression prevalence according to participants’ characteristics and health literacy. Depression prevalence was notably higher among individuals aged ≥65 (19.8%, χ^2^ = 269.74, *p* < 0.001), females (22.2%, χ^2^ = 1082.85, *p* < 0.001), those without a spouse (25.8%, χ^2^ = 1388.57, *p* < 0.001), urban residents (19.9%, χ^2^ = 170.85, *p* < 0.001), individuals with education below middle school (21.1%, χ^2^ = 634.80, *p* < 0.001), unemployed individuals (23.9%, χ^2^ = 1619.15, *p* < 0.001), recipients of the national basic living security program (37.2%, χ^2^ = 1337.02, *p* < 0.001), those with unmet healthcare needs (42.0%, χ^2^ = 1491.06, *p* < 0.001), those reporting poor subjective health (34.8%, χ^2^ = 6684.23, *p* < 0.001), individuals experiencing stress (45.15%, χ^2^ = 7003.80, *p* < 0.001), and those with health literacy scores <3 (24.3%, χ^2^ = 1363.82, *p* < 0.001).

Logistic regression analysis results, as shown in Table 4, revealed that the model had an explanatory power (Nagelkerke’s R^2^) of 0.26 and a significant fit (Wald F = 26,156.50, *p* < 0.001). The risk of depression was significantly higher for females (OR = 1.28, 95% CI: 1.22–1.34), individuals without a spouse (OR = 1.47, 95% CI: 1.40–1.54), urban residents (OR = 1.44, 95% CI: 1.37–1.50), unemployed individuals (OR = 1.47, 95% CI: 1.41–1.54), recipients of the national basic living security program (OR = 1.50, 95% CI: 1.40–1.62), individuals experiencing stress (OR = 4.56, 95% CI: 4.36–4.77), those with fair subjective health status (OR = 1.74, 95% CI: 1.63–1.90), and those with poor subjective health status (OR = 4.33, 95% CI: 4.05–4.63).

## 4. Discussion

This study offers several novel contributions to the understanding of health literacy and its impact on depression among individuals with chronic conditions, particularly in the South Korean context. The findings of this study revealed a significant association between health literacy and the risk of depression among individuals with specific chronic diseases, such as diabetes and hypertension. Lower health literacy levels were observed in the depression risk group. After adjusting for various control factors, the analysis revealed a statistically significant association between health literacy and depression. This aligns with previous research indicating that higher health literacy abilities contribute to better prevention and management of depression [13,20]. Other studies have also reported a negative correlation between health literacy levels and depression, emphasizing the importance of improving health literacy to lower the risk of depression among patients with hypertension and diabetes [12].

The study highlights that individuals diagnosed with both diabetes and hypertension in South Korea have lower health literacy levels and a higher prevalence of depression compared to those diagnosed with only one condition. This co-occurrence’s impact on mental health has not been as extensively studied in South Korea compared to other nations, adding regional specificity to the results. Managing both chronic conditions simultaneously presents more complexities, possibly due to South Korea’s healthcare utilization patterns and the mental health stigma prevalent in the country [21,22].

Additionally, research has indicated that individuals with low health literacy may have difficulty understanding their health status, engaging in adequate self-care practices, and seeking timely medical attention when experiencing symptoms. Moreover, they may be less likely to utilize preventive healthcare services, exacerbating the management of chronic illnesses [23,24]. Furthermore, studies have suggested that improving health literacy can serve as an empowering tool, enabling individuals to access education about mental health services and effectively utilize available resources [13].

Therefore, it is imperative for healthcare providers and policymakers to prioritize efforts aimed at enhancing health literacy among patients with hypertension and diabetes. By improving health literacy levels in these individuals, we can potentially mitigate the impact of depression and enhance overall mental well-being, ultimately leading to better health outcomes and improved quality of life.

Given the high suicide rate and prevalence of depression in South Korea, addressing mental health issues and implementing effective intervention measures become crucial [11]. Encouraging individuals to proactively address their mental health concerns can enhance access to mental health services and promote mental health prevention and promotion initiatives. However, the low utilization rate of mental health services in South Korea highlights the presence of negative prejudice, misunderstanding, and social stigma concerning mental health, making it necessary to develop educational programs and information campaigns to combat these barriers [11,25]. The results of this study can serve as valuable foundational information for designing effective mental health intervention plans for chronic patients.

While demographic and sociological characteristics can have some influence on health literacy, they do not fully determine an individual’s health literacy level [25]. Therefore, tailored intervention strategies aimed at improving health-related knowledge and understanding levels can be beneficial, particularly for groups with inadequate health literacy abilities [26]. Chronic patients may face challenges in improving their health literacy due to various environmental factors, including physical health and economic conditions, necessitating active support at both the community and national levels. By enhancing health literacy among chronic patients, it becomes possible to prevent depression and promote a better understanding of health information.

This study has several limitations that should be acknowledged. First, the cross-sectional design of the study restricts the ability to draw causal inferences between variables. Future research should consider adopting longitudinal approaches to better account for potential time effects and to establish causality. Second, while the study utilized a straightforward method to assess health literacy using two questions from the KCHS, this approach has inherent limitations. The assessment was based on self-reported measures, focusing only on the understanding of oral and written health information. This narrow scope does not capture the full complexity of health literacy, which includes additional skills such as effective communication, navigating the healthcare system, and applying health knowledge in decision-making processes.

Moreover, the validity and reliability of these two questions as a comprehensive measure of health literacy were not explicitly examined within this study. This omission may limit the generalizability of the findings, as the measure may not fully represent the broader concept of health literacy. Future studies should incorporate more robust and validated instruments to assess health literacy, ensuring that a wider spectrum of literacy skills is evaluated. Finally, the study’s reliance on self-reported data for subjective health and other variables introduces potential biases, such as social desirability or recall bias, which could affect the accuracy of the findings.

Despite these limitations, this study utilized data from the 2021 Korea Community Health Survey (KCHS), offering a nationally representative sample of South Korean adults. The use of this comprehensive and recent dataset enhances the relevance of the findings for current healthcare policies in South Korea. While similar associations between health literacy, chronic conditions, and depression may be observed in other countries, the policy implications drawn from this study are specifically tailored to the South Korean healthcare context. This is particularly important for shaping mental health outreach programs and public health campaigns that aim to improve health literacy among South Korean adults [16].

## 5. Conclusions

This study underscores the critical role of health literacy support in reducing the risk of depression among chronic patients. By enhancing health literacy levels among individuals with chronic diseases, it becomes feasible to mitigate the risk of depression and foster a better comprehension of health-related information. Implementing comprehensive health literacy initiatives and cultivating supportive environments are vital steps toward enhancing the overall well-being of individuals grappling with chronic conditions. Such initiatives have the potential to significantly improve mental health outcomes and enhance the overall quality of life for chronic patients.

In conclusion, this study highlights the critical role of health literacy in the prevention and management of depression among individuals with specific chronic diseases, such as hypertension and diabetes. Addressing the challenges associated with low health literacy levels is essential for improving mental well-being and overall health outcomes in this population. Healthcare providers and policymakers should prioritize efforts to enhance health literacy and reduce mental health stigma to ensure better access to mental health services and promote overall health and well-being.

## Figures and Tables

**Table 1:** Characteristics of the participants.

Variable	Category	n (%)
Age	<65	30,949 (40.1)
	≥65	46,184 (59.9)
	mean ± SD	66.91 ± 12.46
Sex	Male	35,835 (46.5)
	Female	41,298 (53.5)
Marital status	With spouse	53,095 (68.8)
	No spouse	24,009 (31.1)
Residential district	Rural	42,211 (53.4)
	Urban	35,922 (46.6)
Education level	≤Middle school	42,558 (55.2)
	>Middle school	34,522 (44.8)
Job	Unemployed	36,188 (46.9)
	Employed	40,943 (53.1)
Recipient of the national basic living security program	Yes	5013 (6.5)
	No	72,108 (93.5)
Unmet healthcare need	Yes	3628 (4.7)
	No	73,361 (95.3)
Subjective health status	Good	19,581 (25.4)
	Fair	34,359 (44.6)
	Poor	23,193 (30.0)
Stress	Yes	15,096 (19.6)
	No	62,037 (80.4)
Health literacy	<3	30,401 (39.4)
	≥3	46,732 (60.6)
	mean ± SD	2.84 ± 0.69
Depression	Yes	13,840 (18.0)
	No	63,149 (82.0)
Chronic disease	Diabetes	10,865 (14.1)
	Hypertension	48,662 (63.1)
	Diabetes and hypertension	17,606 (22.8)

**Table 2:** Depression and health literacy according to chronic disease.

Variable	Category	Chronic Diabetes	Hypertension	Diabetes and Hypertension	*χ* *^2^*	*p*
Health literacy	<3	19,081 (39.2)	3928 (36.2)	7392 (41.0)	98.00	<0.001
	≥3	29,581 (60.8)	6937 (63.8)	10,214 (58.0)		
Depression	Yes	8138 (16.8)	1984 (18.3)	3.718 (21.2)	169.86	<0.001
	No	40,426 (83.2)	8863 (81.7)	13,860 (78.8)		

**Table 3:** Depression according to participants’ characteristics and health literacy.

Variable	Category	Depression		χ^2^	*p*
		Yes	No		
Age	<65	4700 (15.2)	26,217 (84.8)	269.74	<0.001
	≥65	9140 (19.8)	36,932 (80.2)		
Sex	Male	4683 (13.1)	31,095 (86.9)	1082.85	<0.001
	Female	9157 (22.2)	32,504 (77.8)		
Marital status	With spouse	7660 (14.4)	45,374 (85.6)	1388.57	<0.001
	No spouse	6174 (25.8)	17,753 (74.2)		
Residential district	Rural	6696 (16.3)	34,417 (83.7)	170.85	<0.001
	Urban	7144 (19.9)	28,732 (80.1)		
Education level	≤Middle school	8967 (21.1)	33,484 (78.9)	634.80	<0.001
	Middle school	4866 (14.1)	29,622 (85.9)		
Job	Unemployed	8626 (23.9)	27,457 (76.1)	1619.15	<0.001
	Employed	5214 (12.7)	35,691 (87.3)		
Recipient of the national basic living security program	Yes	1857 (37.2)	3137 (62.8)	1337.02	<0.001
	No	11,978 (16.6)	60,005 (83.4)		
Unmet healthcare need	Yes	1524 (42.0)	2104 (58.0)	1491.06	<0.001
	No	12,316 (16.8)	61,045 (83.2)		
Subjective health status	Good	1327 (6.8)	18,238 (93.2)	6684.23	<0.001
	Fair	4467 (13.0)	29,854 (87.0)		
	Poor	8046 (34.8)	15,057 (65.2)		
Stress	Yes	6244 (45.1)	8815 (58.5)	7003.80	<0.001
	No	7596 (12.3)	54,334 (87.7)		
Health literacy	<3	7373 (24.3)	22,947 (75.7)	1363.82	<0.001
	≥3	6467 (13.9)	40,202 (86.1)		

**Table 4:** The effects of health literacy on depression.

Variable (Ref.)	Category	OR *(95% CI **)	*p*
Age (≥65)	<65	1.00 (0.94–1.05)	0.843
Sex (Male)	Female	1.28 (1.22–1.34)	<0.001
Marital status (with spouse)	No spouse	1.47 (1.40–1.54)	<0.001
Residential district (rural)	Urban	1.44 (1.37–1.50)	<0.001
Education level (>middle school)	≤Middle school	1.06 (1.01–1.12)	0.047
Job (employed)	Unemployed	1.47 (1.41–1.54)	<0.001
Recipients of the national basic living security program (no)	Yes	1.50 (1.40–1.62)	<0.001
Unmet healthcare need (no)	Yes	2.32 (2.14–2.51)	<0.001
Subjective health level (good)	Fair	1.74 (1.63–1.90)	<0.001
	Poor	4.33 (4.05–4.63)	
Stress (no)	Yes	4.56 (4.36–4.77)	<0.001
Health literacy (<3)	≥3	0.74 (0.68–0.75)	<0.001

Note: The reference category for each variable is indicated in parentheses. * Odds ratio, ** Confidence interval.

## Data Availability

The datasets generated or analyzed during the study are available from the corresponding author on reasonable request.
